# Omental torsion; an unusual case of acute abdomen. Case report

**DOI:** 10.1016/j.ijscr.2023.107901

**Published:** 2023-01-14

**Authors:** Luis Miguel Carrillo, José de Jesús Marín-López, Oscar Díaz-Barrera, Jesús Alejandro Olvera-Rodríguez, Laura Yazmín Gutiérrez-Gutiérrez, Jacaranda Herrera-Gutiérrez

**Affiliations:** a Department of General Surgery, Hospital General Tercer Milenio, Aguascalientes, Mexico; b Department of General Surgery, Hospital General de Zona 1, Villa de Álvarez, Colima, Mexico; c School of Medicine, Mexico American University of the North, Reynosa, Tamaulipas, Mexico; d School of Medicine, Autonomous University of Aguascalientes, Aguascalientes, Mexico; e School of Medicine, Benemerita Autonomous University of Puebla, Puebla, Mexico

**Keywords:** Omental torsion, Acute abdomen, Laparoscopic surgery, Case report

## Abstract

**Introduction and importance:**

Omental torsion as a cause of acute abdominal pain is extremely rare and difficult to diagnose preoperatively given the non-specific clinical picture.

**Presentation of case:**

We report the case of an adult male who went to the emergency room due to abdominal pain, presented clinical symptoms and laboratory findings consistent with acute appendicitis but was diagnosed intraoperatively with omental torsion and associated necrosis, which was successfully treated by laparoscopic omentectomy.

**Discussion:**

It is a rare entity with a low incidence. The symptoms of the cases reported in the literature are usually confused with other abdominal pathologies such as appendicitis or cholecystitis, so preoperative diagnosis continues to be a challenge. The treatment of choice is the laparoscopic approach, since it allows confirming the diagnosis, evaluating the severity of the ischemia, and ruling out other surgical pathologies.

**Conclusion:**

It is important to consider omental torsion as another differential diagnosis of acute abdomen, which can be satisfactorily resolved via laparoscopy, thus avoiding the development of complications associated with its natural evolution.

## Highlights

• Omental torsion is a rare cause of acute abdominal pain with an incidence of 0.0016% to 0.37%.
• The ambiguity of the symptoms makes it a difficult preoperative diagnosis to perform, the probability is from 0.6% to 4.8%.
• It has been proposed that in unclear diagnosis or when conservative treatment fails, laparoscopy should be performed.
• Laparoscopic omentectomy is the treatment of choice.

## 1 Introduction

First described in 1899 by Eitel, torsion of the greater omentum is a rare cause of acute abdominal pain with an incidence of 0.0016 % to 0.37 % and is the cause of 1.1 % of cases of abdominal pain, so there is little literature about it. The ambiguity of the symptoms makes it a difficult and unusual preoperative diagnosis to perform, the estimated probability is from 0.6 % to 4.8 %, and it can easily be confused with other pathologies [1], [2], [3], [4]. Next, we describe the case of a male patient who presented with clinical symptoms and laboratory findings consistent with acute appendicitis but was diagnosed intraoperatively with omental torsion and associated necrosis. This patient was managed in a public healthcare system setting. This case report has been reported in line with the SCARE 2020 criteria [5].

## 2 Presentation of case

**Fig. 1:**
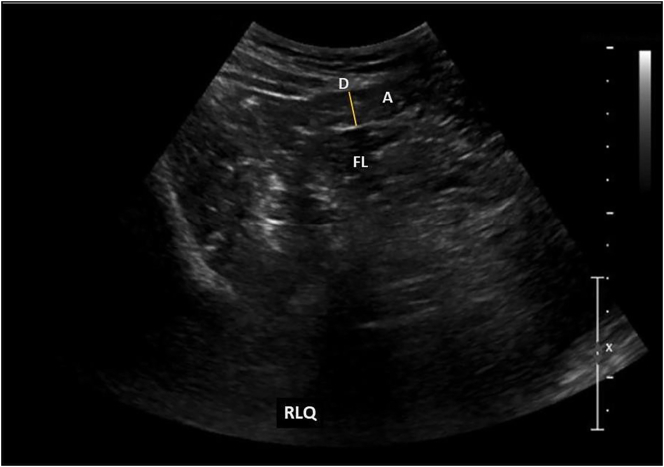
Abdominal ultrasound. RLQ: right lower quadrant. A: appendix. D: 6 mm outer diameter appendix. FL: fluid collection.

**Fig. 2:**
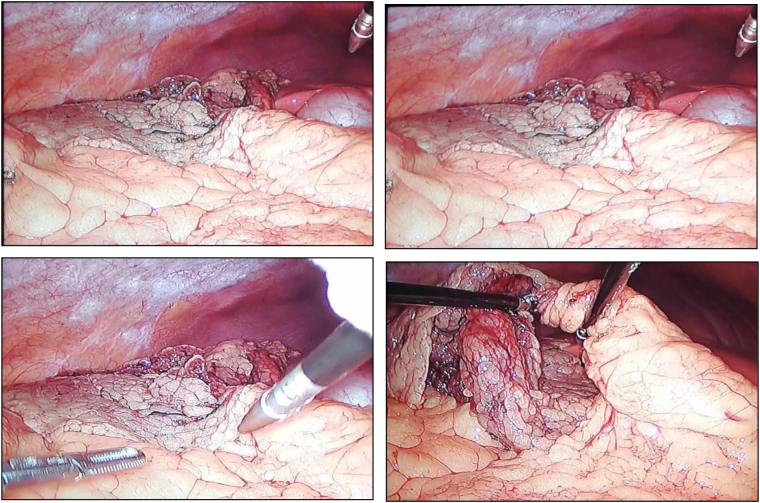
Diagnostic laparoscopy: torsion of the greater omentum in its axial plane with associated distal necrosis.

A 24-year-old male with no significant medical history, went to the emergency room due to abdominal pain, located in the iliac fossa and right flank, which increased with body movements, without irradiation, without other symptoms and with 17 h of evolution. During abdominal physical examination, decreased peristalsis was found, tympanic percussion plus Mc Burney, Blumberg, psoas, obturator and talopercussion signs were positive. Negative bilateral Giordano. Laboratory studies reported hemoglobin of 15.3 g/dL, hematocrit of 46.7 %, platelets 385,000 10^3^/μL, leukocytes 11.08 10^3^/μL, neutrophils of 66 %, normal coagulation times, normal urinalysis. We performed an abdominal ultrasound that showed: aperistaltic, non-compressible, 6 mm outer diameter appendix, little periappendiceal fluid collection and no other findings (Fig. 1). With 20 h of evolution laparoscopic surgical treatment was proposed due to the initial suspicion of acute appendicitis, which is performed by general surgeon in charge of the patient. During the initial laparoscopy, non-inflammatory appendix was observed but a distal segment of the omentum with torsion on its axial axis and secondary necrosis was found (Fig. 2). Said segment was resected with advanced bipolar energy, the extraction bag was introduced, extracting the piece through the umbilical port. The abdominal cavity was explored without identifying other findings, the appendix wasn't resected and the procedure was finished. The patient was discharged 24 h after the surgical event in adequate general conditions and without post-surgical complications. Outpatient follow-up was performed for one month, showing adequate evolution and no late complications.

## 3 Discussion

First described in 1899 by Eitel. However, Bush is credited with the first reported case in 1896. After Bush's description, fewer than 300 cases have been reported in the literature, of which fewer than 26 were treated by laparoscopic surgery. Torsion of the greater omentum is a rare cause of acute abdominal pain with an incidence of 0.0016 % to 0.37 % and is the cause of 1.1 % of cases of abdominal pain [2], [3].

Omental torsion occurs when a segment of it is rotated about its axial axis, becoming strangulated; thus, compromising arterial blood flow, causing necrosis, production of serosanguineous fluid and finally, peritonitis [2], [3], [6], [7]. In most cases, omental torsion occurs between the fourth and fifth decades of life. Men and women have a 5:1 ratio in terms of susceptibility. Children represent only 0.1 % of documented cases [4].

This condition has two different clinical presentations. The primary or unipolar omental torsions and the secondary or bipolar ones. The primaries are associated with anatomical variants of the omentum such as tongue-shaped projections, bifid or accessory omentum, and narrow omental pedicles. These encourage it to rotate, in addition obesity is a factor that influences the abnormal distribution of fat in the omentum, which generates greater weight, and greater risk of torsion. This variant is rare, occurring in only one third of cases [2], [6], [8], [9], [10].

The most frequent type of omental torsion is secondary omental torsion, which is characterized, unlike its counterpart, by bipolarity. This indicates that both ends of the omentum are fixed, both distally and proximally. The distal end of the omentum is associated with scars from previous surgeries or trauma, as well as with inflammation, cysts, tumors, hernias, and other intra-abdominal pathologies that can lead to adhesion formation [6], [7], [8], [9].

Since we could turn a different number of times, the torsion can affect the entire omentum or just part of it. In most cases, omental torsion occurs in the greater omentum, although it has also been reported that it can develop in the lesser omentum [10], [11].

On the other hand, all causes of increased abdominal pressure can be triggers in both primary and secondary types. Some of these causes are copious meals, physical activity, sneezing, coughing, recent abdominal surgeries or trauma, sudden changes in body position, increased peristalsis, and pregnancy [2].

As for the clinical picture, it is characterized by sudden, continuous, non-radiated abdominal pain that worsens over time. The most frequent location is the lower right quadrant of the abdomen, although this in turn will depend on the quadrant where the torsion is generated. Because intestinal peristalsis is not impaired, gastrointestinal symptoms such as anorexia, nausea, and vomiting are absent in more than 50 % of patients. May be accompanied by fever, palpable abdominal mass depending on the extent of the affected segment. Although most patients have only one episode of abdominal pain, repeated pain may indicate intermittent twisting [2], [4], [6].

In addition, leukocytosis is present on laboratory studies. Since the symptoms are nonspecific, and since it is a rare disease, the most common differential diagnoses are acute appendicitis, acute cholecystitis, acute diverticulitis, peptic ulcer disease or infarction of an epiploic appendix. In women of reproductive age, salpingitis, torsion of the ovarian cyst and ectopic pregnancy are the main differential diagnoses [1], [3], [6], [12].

Therefore, the probability of making the diagnosis of this entity in the preoperative moment is between 0.6 and 4.8 %. Currently, high-quality computerized axial tomography (CT), as well as ultrasound (USG), have made it possible to make the diagnosis preoperatively [8], [11].

In the USG, peritoneal fluid can be found, as well as an ovoid mass adhered to the anterior abdominal wall. This mass is described as an incompressible hyperechoic lesion with hypoechoic borders that is related to the hypersensitivity of the lesion. On CT, an ovoid mass adhered to the abdominal wall in the umbilical or anterolateral region, in the middle third of the transverse colon, has been described, as well as hardening and strands of omental fat.

Two characteristic signs can be seen:

1. Vascular pedicle sign: the mesenteric vessel is surrounded by multiple turns of the smaller branches of the mesenteric artery.
2. Pinwheel sign: cloudy mass of fat with concentric lines, with twisting of blood vessels within the greater omentum. This turns around the central vascular line, this last sign confirms the torsion of the greater omentum [3], [4], [7], [8].

Magnetic resonance shows hypointense linear structures corresponding to low flow in the mesenteric vessels within a hyperintense mass of fat on T1. While in T2, congestion and edema of the greater omentum are observed, as well as a hyperintense tumor [4], [13].

There are 2 basic approaches to treating omental torsion: conservative and surgical. When the diagnosis is made with radiological techniques, conservative treatment can be proposed as the first line, with surveillance during the first 24–48 h. Such treatment consists of the administration of oral analgesics, anti-inflammatory drugs and prophylactic antibiotics. Conservative treatment may fail and lead to abscess formation and intra-abdominal adhesions, which can be dangerous for the patient. The main argument against conservative treatment is the failure to identify an inflammatory condition in the appendix, which could expose the patient to complications such as peritonitis and intra-abdominal abscess formation. Therefore, it has been proposed that in the face of an unclear diagnosis or when conservative treatment fails, laparoscopy should be performed [3], [4], [8], [11], [14].

With the introduction of laparoscopy and its widespread use, omental torsion or infarction can now be easily seen, and the chances of missing pathology during surgery have decreased significantly. Surgical treatment consists of removing the injured area of the omentum, since there may be necrotic omental tissue, which various authors consider prevents sepsis, as well as allowing a shorter hospital stay. Laparoscopy allows for a confirmatory diagnosis, evaluation of the severity ofdistal ischemia of the omentum due to torsion, as well as omental resection or detorsion of the greater omentum. In most cases, intraoperative conversion to laparotomy is not required since evacuation of the resected specimen can be accomplished through the trocar sites [4], [7], [12].

In open surgery, diagnosis is likely to be missed as exploration is difficult through a McBurney-type incision, or any infraumbilical incision, used for standard appendectomy [15]. Laparoscopy has several advantages, including a complete examination of the abdominal cavity to confirm the diagnosis and the advantages of minimally invasive surgery, such as reduced postoperative pain and wound- related complications. Clinical signs and symptoms disappear immediately after resection of the affected portion. Laparoscopic omentectomy is the treatment of choice [1], [3], [16].

## 4 Conclusion

It is important to consider omental torsion as another differential diagnosis of acute abdomen, which can be satisfactorily resolved via laparoscopy, thus avoiding the development ofcomplications associated with its natural evolution.

## Patient consent

Written informed consent was obtained from the patient for publication of this case report and accompanying images. A copy of the written consent is available for review by the Editor-in-Chief of this journal on request.

## Ethical approval

The study is exempt from ethnic approval by the institution.

## Sources of funding

N/A.

## Author contribution

Writing the paper and assisting in the procedure: Luis Miguel Carrillo, José de Jesús Marín-López.

Performing the procedure and assisted in literature search: Luis Miguel Carrillo, José de Jesús Marín-López, Oscar Díaz-Barrera, Jesús Alejandro Olvera-Rodríguez.

Assisted in writing manuscript: Luis Miguel Carrillo, José de Jesús Marín-López, Oscar Díaz-Barrera, Jacaranda Herrera-Gutiérrez.

Data/evidence collection: Luis Miguel Carrillo, Jesús Alejandro Olvera-Rodríguez, Laura Yazmín Gutiérrez-Gutiérrez.

Designed the study: Luis Miguel Carrillo, José de Jesús Marín-López, Jesús Alejandro Olvera-Rodríguez, Laura Yazmín Gutiérrez-Gutiérrez.

Review of the manuscript: Luis Miguel Carrillo, José de Jesús Marín-López, Oscar Díaz-Barrera, Jacaranda Herrera-Gutiérrez.

Literature Review and guarantor: Luis Miguel Carrillo, José de Jesús Marín-López.

## Guarantor

Luis Miguel Carrillo.

José de Jesús Marín-López.

## Research registration number

N/A.

## Declaration of competing interest

N/A.
